# Supplementary material for: Colostrum provision and care of calves among smallholder farmers in the Kaziranga region of Assam, India
Source: PLoS One. 2020 Mar 11;15(3):e0228819. doi: 10.1371/journal.pone.0228819 (PMC7065800; doi:10.1371/journal.pone.0228819)
Supplement: S1 Data — (DOCX) [file pone.0228819.s001.docx]

**1**

Date: Time: Interviewer: AH Translator: Jadumomi Goswami

Location/ who else is present / what else is happening?

Home, porch

Name: Male/ ~~female:~~ Age: 45

**Your family and household**

**Who lives in your house?**

(Can we meet them please? What do they do? What do you want to do when grown up?)

Wife, Son (16), Son (13)

Would like sons to get good government job or army job (can’t afford higher education) must gain skills. If not, farming

**How is your family/ household supported?** (food, money, rent, etc).

Farming (paddy, mustard). Labouring

**What animals does your family/ household have? Why are your animals important?** (Can we see your animals please? Why do you keep them? How do animals help to support your household? )

2 draft ox. 6 cows- 5 milk cows but only one in milk now. 2 calves. (where is missing cow?) Sometimes sell calf at 2 y/o.

2 goats- sell goats, don’t eat. 3 chickens. (ducks died- sick after flood)

**What is a ‘good’ animal?** How do you know?

Ox: size of thighs

Cow; length, belly size, udder (plump- good, flat no milk)

Look not important

**What things limit how much your animals give?** ( provide for your household?)

Tiger last loss Dec 2016 cow killed tied up in paddy, seen by neighbour. Attack on cow in shed in 2000. Killed, tiger seen dragging away, then dropped.

Other wild animals.

**Young Animals**

Where do you keep your calves? Can we see please?

**How many of your cows/ buffs/ goats had a baby in the last year?**

5 cows, 4 gave birth

**Where are those baby animals now?**

1 dead flood

1 dead BQ

2 alive and at home

**When a calf is born, can you teach me about the first day of its life?**

(When should it first drink milk (colostrum)? How much? What can you do to help it to be healthy? Who helps it?)

Tie rice straw in mouth and round back of neck/ behind ears (then check suck) leave in place until breaks (approx. 30 minutes) or mother removes. Belief is it helps to drink.

Colostum (‘Phea- hom) when placenta falls (usually 1-2 hrs) milk off colostrum- 0.5 – 1 L varies with cow size. Remaining colostrum calf allowed to drink. Make colostrum sweets and eat.

No other treatment to calf.

Cow not milked again until calf 7 days old.

**How much milk does an older calf drink and how often does it get it?** (Can you teach me how you do this? Who is responsible for this?)

Milk in morning only, calf gets rest. Used to milk twice daily.

(for native cattle) Calf separated from cow overnight. Send calf to cow- starts let down- remove calf- milk cow empty– return calf – second let down – remove calf – milk again – calf gets rest of milk.

**Do your calves have any food apart from milk? Water?**( Where? How often? Who’s job?)

Calf drinks milk and eats grass from 1 month. Water bowl.

All family members do, whoever available.

**How much milk does your family get each day from your animals?** (What do you do with it? When do you first milk a cow/ buff /goat for drinking?)

Milk 1 – 2 L/ cow daily, dependant on cow size. Sufficient for family. Was selling 1L daily, now supply not enough to sell (since two dead calves).

**Adult Animals**

**Can you show me where your animals rest in the day? At night?**

Morning tie in paddy, evening feed and graze at paddy side, home in evening. Cooked food at dawn- rice husk, banana stem, salt, stay in shed overnight.

During flood, bring rice straw and cut grass.

Taken to river or water hole at paddy twice daily. Bowl of pump water at night.

**How do your animals get food? What**? (Who (family member) is responsible for this? Can we meet them please? How much land do you have? Can we see it?) **Where do your animals drink? How often?**

**Did you vaccinate your animals last year? Why / why not?**

No. Dr didn’t come.

Don’t know what to give. Don’t think about giving medicine to an animal which is not sick.

**Do you de-worm animals? How? Why/ why not?**

No. Sometimes see worms in dung- give sugar cane leaf.

Leaches attack navel in river- feed sugar cane leaf and mustard oil and camphor in wound (effective for leaches and flies)

**Do you do anything else to help your animals?**

**You live close to the forest reserve. Does this affect you?**

Elephant, rhino, buffalo, deer, boar destroy crops. Respondant spends every night in growing season in tungi, when crops ripe- many people in tungi. Use fire, shouting, torches, crackers from FD if they have them.

**Animal health education**

**Is there anyone who can treat your animals?** (What would you like the vet doctor to know more about?)

Vet doctor 5km, doesn’t usually come to village/ slow to attend, no one in village.

**Would you like to learn more about animal health? Why?**

Yes, save own cattle. Cow Rs 15 -17k, Ox Rs 22 – 25K

**What problems are there for people trying to learn about animal health in your village?** ( Why? Which ways of learning about animal health would be good for you (village meetings, classes, leaflets, radio broadcasts, internet information, etc)?)

No guidance when young, keen to learn. Wants 10 days training, then books- can someone provide this?

**What changes could make your farm to more productive/ better?**

Learning. Paddy is main enterprise so cattle get less attention.

**What do you think is the future of this farm? (**Who will you pass your knowledge of farming on to?)

Want to increase size of farm, buy cattle, buffalo, been planning for 8 years but paying for sons education and unable to afford both. If bigger farm, would not be able to labour for cash. Prefer farming, as getting older it is satisfying to grow farm.

Sons? If they get good jobs he will continue to look after farm. Dream is to buy land on high ground that won’t flood. Won’t have to take cattle to the road during the flood.

**Anything else you would like to tell us?**

Vet services would be a big morale booster. TCF vaccine programme protected animals, can we get this help for other problems. The medicine for this came from very far, we don’t have resources for that and I am very grateful to TCF for this.

Can 2 or 3 youths from our village be trained in basic veterinary care?

What about compensation policy from FD for crop damage? NP explained.

**2**

Village: Date: Time: Interviewer: Andy Translator: Jadu

Location/ who else is present / what else is happening?

Home. Wife and mother present – asks them a lot, esp re illness

Name: Male/ ~~female:~~ Age: 49

**Your family and household**

**Who lives in your house?**

(Can we meet them please? What do they do? What do you want to do when grown up?)

Self

Wife

Son 23 learning to drive JCB

Son 20 Helping farm

**How is your family/ household supported?** (food, money, rent, etc).

Farming: paddy veg for house

Labour before, now stop to concentrate on farm

**What animals does your family/ household have? Why are your animals important?** (Can we see your animals please? Why do you keep them? How do animals help to support your household? )

Cow 2, 1 in milk

Calf 2, sell if money problem- usually happens (use like bank)

Goat 4 sell, don’t eat

Duck 2 eat eggs

Ox 2 draft

**What is a ‘good’ animal?** How do you know?

Ox: thigh muscle, body size, hump size

Cow body size, udder confirmation

Avoid unusual signs (features?)

Appearance not important but religion (superstition?) says black goat has more meat and better flavour

**How do you know if an animal is healthy?** How do you know if an animal is unhealthy?

Poor movement, not drinking water

**What things limit how much your animals give?** ( provide for your household?)

Nothing

Wife treats sick animals and milks cow

**Young Animals**

Where do you keep your calves? Can we see please?

**How many of your cows/ buffs/ goats had a baby in the last year?**

2 cows, 1 calf 1 alive and well,

Another calf 1.5 years old

**Where are those baby animals now?**

**When a calf is born, can you teach me about the first day of its life?**

(When should it first drink milk (colostrum)? How much? What can you do to help it to be healthy? Who helps it?)

Navel care- wash with mustard oil for 5-6 days and remove flies. Himax if flies come. Wash calf after birth (mustard oil and cloth), keep warm.

Colostrum: milk off 0.5 – 1L varies with cow size. Put calf on tit and leave to drink. Wife in charge.

Next milking 15 days

**How much milk does an older calf drink and how often does it get it?** (Can you teach me how you do this? Who is responsible for this?)

Calf full drinking for 15 days

Milk once daily, morning, all 4 teats, not empty

**Do your calves have any food apart from milk? Water?**( Where? How often? Who’s job?)

Grazing, drink on own (water?).

Cow tied in paddy, calf loose.

**How much milk does your family get each day from your animals?** (What do you do with it? When do you first milk a cow/ buff /goat for drinking?)

1. 1.5L / day. Sell 1L

**Adult Animals**

**Can you show me where your animals rest in the day? At night?**

Day: tied in paddy, night shed

**How do your animals get food? What**? (Who (family member) is responsible for this? Can we meet them please? How much land do you have? Can we see it?) **Where do your animals drink? How often?**

Salt water once daily

During flood: rice straw, cut grass, banana tree (whole tree, chopped ) w/ rice husk.

1 banana tree lasts 2 days. Tree grows in 6- 12 months, plant every year.

**Did you vaccinate your animals last year? Why / why not?**

Since 2015. Done by State Animal Husbandry Dept. Did not know about vaccination before that. Will continue.

**Do you de-worm animals? How? Why/ why not?**

No. Sometimes see worms in cow dung. Animal recovers.

**Do you do anything else to help your animals?**

**You live close to the forest reserve. Does this affect you?**

Crop raiding: Elephant, rhino. Elephants eat banana trees and sometimes injure people.

Tiger: killed cow in shed 3-4 years ago

**Animal health education**

**Is there anyone who can treat your animals?** (What would you like the vet doctor to know more about?)

No

**Would you like to learn more about animal health? Why?**

“I don’t have time, but my sons can learn”

**What problems are there for people trying to learn about animal health in your village?** ( Why? Which ways of learning about animal health would be good for you (village meetings, classes, leaflets, radio broadcasts, internet information, etc)?)

Sons should read books first, then go for practical training.

**What changes could make your farm to more productive/ better?**

“There is no money. If there was money I could consult the doctor more often.”

**What do you think is the future of this farm? (**Who will you pass your knowledge of farming on to?)

“I want to increase the number of animals as land for cultivation limited. If I buy in new breeds of cows then milk production could be increased.”

**Anything else you would like to tell us?**

“Protecting crops from tungi is hard work and I am up all night. The solar fence does not protect us from crop raiding. I have no cultivation land of my own. I work another man’s land and give him 50% of the crop. Crop raiding is a village problem.”

AH: “Is crop raiding more or less since the fence was put up?”

“Yes, crop raiding is less since the fence was built. The fence is helpful. Crop raiding is less this year. Our main problem is the flood. Now we are cropping in a different season, but this means we are increasing spending on water."

**3**

Village: Date: Time: Interviewer: Andy Translator: Jadu

Location/ who else is present / what else is happening?

Home, Mother present and very knowledgeable

Name: ~~Male~~/ female: Age: 30

**Your family and household**

**Who lives in your house?**

(Can we meet them please? What do they do? What do you want to do when grown up?)

Self

Younger brother : driver

Mother

Sister in law

Nephew: 3 yr

Uncle

Uncle wife

Son 15 years

Son 13 years

Daughter 10 years

**How is your family/ household supported?** (food, money, rent, etc).

Farming: paddy, mustard veg for house, occasional sale

Weaving and weaving trainer

Trading goods: store, farming goods. Own one car: transport goods and other driving

**What animals does your family/ household have? Why are your animals important?** (Can we see your animals please? Why do you keep them? How do animals help to support your household? )

Cow: 2, 1 in milk

Calf: 3 , may sell if males (or sell own Ox if old and train new). Use all calves.

Goat 12 - 13 sell, don’t eat (vegetarian)

Ox 2 draft

**What is a ‘good’ animal?** How do you know?

Cow: hair short and soft, navel slightly long- gives more milk

Ox: teeth

No black cows (“Black cows don’t suit this house”), only brown.

**How do you know if an animal is healthy?** How do you know if an animal is unhealthy?

Not eating (“main sign of illness”), Mood.

Less problems since started vaccinating.

**What things limit how much your animals give?** ( provide for your household?)

Tiger kills goats and cows. Took a goat from shed 4 years ago- saw pug marks.

Saw leopard in tree above shed 15 days ago.

**Young Animals**

Where do you keep your calves? Can we see please?

**How many of your cows/ buffs/ goats had a baby in the last year?**

2 cows, 2 calves 6-7 mths/o

**Where are those baby animals now?**

**When a calf is born, can you teach me about the first day of its life?**

(When should it first drink milk (colostrum)? How much? What can you do to help it to be healthy? Who helps it?)

Clean cow and calf.

Tie rice straw through mouth (10 -15 mins) and check movement of tongue – claf or mother break straw.

Cut tips off hooves- prevent big hoof problem,, goat same.

Colostrum (“Phae- Hoo”) : milk out 70%, leave rest for calf (approx. 1L house, 0.5L calf). Make sweets.

Next milking 7d, cow and calf together at all times until then.

**How much milk does an older calf drink and how often does it get it?** (Can you teach me how you do this? Who is responsible for this?)

Calf drinks for a few minutes- let down – remove calf- milk 2 teats empty- rest for calf.

When calf 3 m/o- milk twice daily

**Do your calves have any food apart from milk? Water?**( Where? How often? Who’s job?)

From 3 months grazing and cooked food (veg, banana tree, rice husk)

“Sicora” tick problems and leaches: “How do we get rid of these? I realise ticks are important for the cycle of life, they provide food for the birds and small creatures, but they feed on our animals. I use medicine that I pour along the calf’s backbone, but it makes the calf weak. Is there another way to deal with them?”

**How much milk does your family get each day from your animals?** (What do you do with it? When do you first milk a cow/ buff /goat for drinking?)

Once daily milking 2.5 L/cow Twice daily 3.5 L/cow Use 1L daily, sell surplus.

Jersey cross cows, once had a cow that gave 5L morning and 2L evening

**Adult Animals**

**Can you show me where your animals rest in the day? At night?**

Shed at night, tied in paddy in day- no shade in paddy, if too hot take to shade of tree.

**How do your animals get food? What**? (Who (family member) is responsible for this? Can we meet them please? How much land do you have? Can we see it?) **Where do your animals drink? How often?**

Grazing, cooked food at night.

Water taken to field at noon, very hot day- take 2-3 times. Rice water/ rice washing water w/ salt added. All family members do.

**Did you vaccinate your animals last year? Why / why not?**

Yes. 2015, 2016. Prevents Dz. Occurs d/t Forest Dept initiatives to protect wild animals. Consultation of Forest Dept and Eco Development Committee in order to take advantage of Dz prevention initiatives. I have seen animals being vaccinated at times for my whole life.

**Do you de-worm animals? How? Why/ why not?**

Yes. Dr medicine or pharmacy shop

“Mackie pilou” (maggots) wash wound and apply petrol, kerosene or turpentine. Use Himax on wounds to prevent.

“Ouja”- faith healer (not a priest)- pray and perform a ritual then give something in a wrap of banana leaves which the animal must eat. Treats maggots. Is it effective?- not sure.

**Do you do anything else to help your animals?**

Use smoke to prevent mosquitos in shed or mosquito nets

Nose rope placed at 5 – 7 years. Make animal lay down (cast) tie all legs, piece nose w/ bamboo spike and then use it to draw rope through.

**You live close to the forest reserve. Does this affect you?**

Spread of Dz from wild animals.

Crop raiding- elephant “hati”(biggest problem), also wild boar “guar-ri”, deer “mus-tad”, wild buffalo “mo-ho”. Rhino “gore” less often.

**Animal health education**

**Is there anyone who can treat your animals?** (What would you like the vet doctor to know more about?)

Vet Dr 5km away. “Not very expert. One buffalo died from wtong treatment a few years back. Buffalo had Dz, I told the Dr and he vaccinationed the buffalo anyway and it died. This was years ago”.

**Would you like to learn more about animal health? Why?**

Yes. “ I didn’t learn when I was young as my father and brother were alive and they did those things. Now I need to know more as I am running the house.”

**What problems are there for people trying to learn about animal health in your village?** ( Why? Which ways of learning about animal health would be good for you (village meetings, classes, leaflets, radio broadcasts, internet information, etc)?)

I have some time available. “Learning theory from books is good, but I need practical training so that I can really learn to do these things.”

**What changes could make your farm to more productive/ better?**

I need help or money before I can make changes. I would like to buy more cows but then I would have to hire more men to help look after them. I did a training course, Dairy Development Trainin.

**What do you think is the future of this farm? (**Who will you pass your knowledge of farming on to?)

I want to expand

I don’t know (who I will pass my knowledge on to).

**Anything else you would like to tell us?**

“It would be good if you (Andy) did some training in the days while you are here. It would be very helpful ti us (the villagers)”

**4**

Village: Date: Time: Interviewer: Andy Translator: Jadu

Location/ who else is present / what else is happening?

Home. Mother present and very knowledgeable

Name: Papul Bora Male/ ~~female~~: Age: 27

**Your family and household**

**Who lives in your house?**

(Can we meet them please? What do they do? What do you want to do when grown up?)

Self: Driver also

Father: 50

Mother: 45 All working farm

Sister: 17

Brother: 21

**How is your family/ household supported?** (food, money, rent, etc).

Farming: paddy, mustard, potato veg for house, occasional sale

Driving

**What animals does your family/ household have? Why are your animals important?** (Can we see your animals please? Why do you keep them? How do animals help to support your household? )

Cow: 3, milk and dung for cultivation

Calf: 2, sell if males at 8 – 10 mth/o, keep females.

Goat 4, sell, eat at festivals occasionally

Ox 0. Sold- old and sick (15 yr/o). Sold in market. Currently rent tractor (cultivation machine) when required, costs Rs300 / bihar (unit of agricultural land). Intend to buy more Ox before flood so don’t have to rent tractor.

Chicken 10 – 15. Sell, Also eat.

**What is a ‘good’ animal?** How do you know?

Ox: Legs, teeth and hooves.

Cow: Feel sometning inside when looking at cow. Also, is it giving a lot of milk? What is the cow eating? Udder and teat size is also important.

“Colour should suit the house. Black and red for this house.” Colour is not main consideration, other things come first.

**How do you know if an animal is healthy?** How do you know if an animal is unhealthy?

“Not eating. Laying down- assume something is wrong. A healthy cow is always active. Eats a drinks a lot.”

**What things limit how much your animals give?** ( provide for your household?)

“Everything is 100%, because everything will occur. We have no problems except Dz.”

**Young Animals**

Where do you keep your calves? Can we see please?

**How many of your cows/ buffs/ goats had a baby in the last year?**

3 cows, 3 calves

**Where are those baby animals now?**

1 died after flood. Not eating after flood finished.

**When a calf is born, can you teach me about the first day of its life?**

(When should it first drink milk (colostrum)? How much? What can you do to help it to be healthy? Who helps it?)

“Phoa- Ho”- milk cow out. Make sweets. Remove 0.5 – 1 L. Yellow colour faded in milk before calf drinks.

Clean calf.

Tie rice straw through mouth (30 mins) – makes calf strong at suckling.

Start fire to warm calf if born in winter.

Next milking 5-6d, cow and calf together at all times until then.

**How much milk does an older calf drink and how often does it get it?** (Can you teach me how you do this? Who is responsible for this?)

Tie calf separate from cow until midnight- loose in morning wait 30 mins, then milk cow. Calf drinks first.

All family members do.

Milk once daily for 15 days, then twice daily

**Do your calves have any food apart from milk? Water?**( Where? How often? Who’s job?)

When starts grazing give cooked food (rice polis and rice husk). Banana tree. In evening, father does.

**How much milk does your family get each day from your animals?** (What do you do with it? When do you first milk a cow/ buff /goat for drinking?)

1. 2L / cow / day. Twice daily = 4L.

**Adult Animals**

**Can you show me where your animals rest in the day? At night?**

Free graze, no tie, shed at night

**How do your animals get food? What**? (Who (family member) is responsible for this? Can we meet them please? How much land do you have? Can we see it?) **Where do your animals drink? How often?**

Grazing, cooked food at night- rice polis and banana tree.

Store fodder for flood time.

Self- drinking at river. Don’t give water at night but water in cooked food.

**Did you vaccinate your animals last year? Why / why not?**

Yes. 2016 first time. Other people told me that it will reduce infections. All kinds of disease.

**Do you de-worm animals? How? Why/ why not?**

No. I don’t see worms.

**Do you do anything else to help your animals?**

Nose rope placed at 5 years. Father does. He lays ox down (cast) and tie all legs, piece nose w/ bamboo spike and then use it to draw rope through.

**You live close to the forest reserve. Does this affect you?**

Elephant and boar destroy crops.

**Animal health education**

**Is there anyone who can treat your animals?** (What would you like the vet doctor to know more about?)

There is a village man with some knowledge, he had Govt training.

**Would you like to learn more about animal health? Why?**

Yes. “The I can help family cattle and neighbours.”

**What problems are there for people trying to learn about animal health in your village?** ( Why? Which ways of learning about animal health would be good for you (village meetings, classes, leaflets, radio broadcasts, internet information, etc)?)

I lack scope of learning (school/ institute) money is also an issue.

Practical learning is best. Meetings are ok. Leaflets are ok, but not my preferred way. Radio can only be a help after practical teaching.

**What changes could make your farm to more productive/ better?**

More cows and feed well for better milk production

**What do you think is the future of this farm? (**Who will you pass your knowledge of farming on to?)

I want to learn about vet care and buy more cattle, ones with better milk production that are easy to handle.

I want to carry on here. I think about it a lot.

**Anything else you would like to tell us?**

“If a few people are trained (as gau sewek) it will be very good for the village. We don’t have to wait for veterinary doctor and can look after cattle in a very good way.”

**5**

Village: Date: Time: Interviewer: Andy Translator: Jadu

Location/ who else is present / what else is happening?

Home, Mother, wife, children. Asks wife a lot.

Name: Male/ ~~female~~: Age: 39

**Your family and household**

**Who lives in your house?**

(Can we meet them please? What do they do? What do you want to do when grown up?)

Self: teacher also (village school)

Wife

Mother:

Son: 13

Daughter: 7 Have own will, I would like them to get good jobs. He likes electronics, she likes dance and music.

**How is your family/ household supported?** (food, money, rent, etc).

Farming: paddy, mustard, veg for house and sale

Teaching (school not fully supported by Govt)

Small shop at house, sell veg and a few other items

**What animals does your family/ household have? Why are your animals important?** (Can we see your animals please? Why do you keep them? How do animals help to support your household? )

Cow: 2, milk and dung for cultivation

Calf: 3, sell when need money (use as a bank) or keep to increase herd. One is older (3yrs) calf.

Goat 4, sell only, vegetarian.

Ox 2. cultivation

**What is a ‘good’ animal?** How do you know?

Ox: Teeth (age), physical figure. Touch body and feel power.

Cow: Body size and heavy backside. And big udder.

For this house, red and white. Not main thing.

**How do you know if an animal is healthy?** How do you know if an animal is unhealthy?

Healthy: horns and legs

Unhealthy: Mood. Laying down. Not eating. Movement

**What things limit how much your animals give?** ( provide for your household?)

Flood

Sufficient fodder not always available

Flies and mosquitos

**Young Animals**

Where do you keep your calves? Can we see please?

**How many of your cows/ buffs/ goats had a baby in the last year?**

2 cows, 2 calves this year, plus one older.

**Where are those baby animals now?**

Here.

**When a calf is born, can you teach me about the first day of its life?**

(When should it first drink milk (colostrum)? How much? What can you do to help it to be healthy? Who helps it?)

Clean calf. Make fire to warm calf in winter. Help to stand.

Milk all 1 litre “yellow milk”“Phaa- Ho”- milking all 4 teats. Take for house. Make sweets. Rest of phaa ho for calf. This is 30- 40% remaining for calf (guess?).

Next milking 5-6d, cow and calf together at all times until then.

**How much milk does an older calf drink and how often does it get it?** (Can you teach me how you do this? Who is responsible for this?)

Milk once daily. Get 1 – 1.5L from milking all 4 teats.

Calf separate from cow overnight. Release calf, after calf drinking for a few moments- remove calf and milk cow

Husband teaching in morning, so other family members responsible.

Cow and calf together in day

**Do your calves have any food apart from milk? Water?**( Where? How often? Who’s job?)

Calf starts grazing after 1 month. Given rice water also. Drinks water with cow.

Everyone’s responsibility.

**How much milk does your family get each day from your animals?** (What do you do with it? When do you first milk a cow/ buff /goat for drinking?)

- 1. L / cow / day. Therefore 3 L daily. Drink 1L, sell 2L

**Adult Animals**

**Can you show me where your animals rest in the day? At night?**

Day- tied in paddy to graze, shed at night. No shade in paddy, if very hot we take to shade.

**How do your animals get food? What**? (Who (family member) is responsible for this? Can we meet them please? How much land do you have? Can we see it?) **Where do your animals drink? How often?**

Grazing paddy.

Cooked food given at night- rice husk and vegetables.

During flood time feed banana tree also.

Water given morning and night, during summer at midday also.

**Did you vaccinate your animals last year? Why / why not?**

Yes. Last 10 years. We o this after the flood.

**Do you de-worm animals? How? Why/ why not?**

De-worming tablets given to calves at 6 – 12 months.

**Do you do anything else to help your animals?**

**You live close to the forest reserve. Does this affect you?**

Crop raiding by elephant and wild boar.

Predation by tigers, though this has never happened to my animals.

**Animal health education**

**Is there anyone who can treat your animals?** (What would you like the vet doctor to know more about?)

No.

**Would you like to learn more about animal health? Why?**

Yes. “I can do this for my own cattle and my community.”

**What problems are there for people trying to learn about animal health in your village?** ( Why? Which ways of learning about animal health would be good for you (village meetings, classes, leaflets, radio broadcasts, internet information, etc)?)

I have had a lack of exposure to this type of learning. I can learn part time but the training must be in a local place. Short training is better for me.

**What changes could make your farm to more productive/ better?**

I want to introduce hybrid animals with better genetics to increase milk production

**What do you think is the future of this farm? (**Who will you pass your knowledge of farming on to?)

We are going along but we are too busy to expand.

My son will probably continue to farm here.

**Anything else you would like to tell us?**

**Interviews 2017**

**6**

**Village**: **Date**: **Time**:

Interviewer: Andy Translator: Jadu

**Location/ who else is present / what else is happening?**

Outside house being built. Mother present, children and other family members coming and going.

**Name**: (and mother) **Male/ ~~female:~~** **Age:** 49

**Your family and household**

**Who lives in your house?**

(Can we meet them please? What do they do? What do you want to do when grown up?)

Self

Mother

Sister

Brother (works for defence service)

Brother wife

Twin daughter: 11 “I want my children to be self- independent and become educated.”

Twin daughter: 11 “The future depends on them.”

Son: 6

Brother daughter: 2

Wife present but not listed by interviewee.

**How is your family/ household supported?** (food, money, rent, etc).

Farming: Paddy, mustard, Peas, Daal. Vegetables

Brother’s salary

Dairy farm (“jerseys”- local expression which appears to mean dairy cattle) at another location.

**What animals does your family/ household have? Why are your animals important?** (Can we see your animals please? Why do you keep them? How do animals help to support your household? )

Cow: 4 milk, calves, dung (biogas made for cooking)

Calf: 0 (There were 2, but they are dead). Keep females to increase cow numbers, sell males

Goat: 13 Sell to butcher. Occasionally family eat one goat if there is a wedding or other event.

Ox: 0

Hire tractor for cultivation. It is difficult to raise ox. One day we will probably buy a tractor

**What is a ‘good’ animal?** How do you know?

Cow: milk veins, facial structure, especially eyes. Udder size.

**How do you know if an animal is healthy?** How do you know if an animal is unhealthy?

Eating style. Style of motion, coat condition

**What things limit how much your animals give?** (provide for your household?)

“Dhar” (specific type of fly): bite cows and causes blood loss (only “jerseys”)

**Young Animals**

Where do you keep your calves? Can we see please?

**How many of your cows/ buffs/ goats had a baby in the last year?**

4 cows, 2 calved

**Where are those baby animals now?**

Dead. 1 sudden death at 6 months, 1 died of 15 days from “weakness”

**When a calf is born, can you teach me about the first day of its life?**

(When should it first drink milk (colostrum)? How much? What can you do to help it to be healthy? Who helps it?)

Clean calf, clean udder, put calf to suck. 1^st^ milk (“Phaa- Ho) calf drinks. Drinks 1 -1 .5L (how do they know this?).Then milk out.

Mustard oil in calf mouth (one tiny cup).

Next milking- next day.

**How much milk does an older calf drink and how often does it get it?** (Can you teach me how you do this? Who is responsible for this?)

In morning calf drinks then we milk the cow. Then the calf roams free for the day and goes back. At evening the calf drinks again. Then cow milked again in the evening.

“If you don’t milk a cow empty then the milk clots in the cow’s udder. If this happens the cow must have an injection (antibiotic?) to prevent illness and the udder milked empty.”

**Do your calves have any food apart from milk? Water?** (Where? How often? Who’s job?)

Grass. Cooked food eaten from one month old: Rice polis, rice husk, vegetables, morning and evening.

Water twice daily.

**How much milk does your family get each day from your animals?** (What do you do with it? When do you first milk a cow/ buff /goat for drinking?)

12 L /day / cow

2 cows in milk, 2 cows pregnant not in milk.

**Adult Animals**

**Can you show me where your animals rest in the day? At night?**

In shed at all times

**How do your animals get food? What**? (Who (family member) is responsible for this? Can we meet them please? How much land do you have? Can we see it?) **Where do your animals drink? How often?**

Rice straw, green folder, rice husk, wheat husk, mineral powder and calcium.

Cooked food w/ water

100L water / day /cow

**Did you vaccinate your animals last year? Why / why not?**

Yes, since I bought in Jerseys. 10 years.

No Soboka in my cows.

**Do you de-worm animals? How? Why/ why not?**

Yes. Goats once per year

Cows. 4 times per year

Tablets from pharmacy.

**Do you do anything else to help your animals?**

Wash daily

Spray for insects on cows

**You live close to the forest reserve. Does this affect you?**

Spread of disease from wild animals to domestic animals.

Everything else is positive for kaziranga.

**Animal health education**

**Is there anyone who can treat your animals?** (What would you like the vet doctor to know more about?)

No. Dr/ compounder in Sabzuri 5km.

**Would you like to learn more about animal health? Why?**

Yes. Improve productivity.

**What problems are there for people trying to learn about animal health in your village?** ( Why? Which ways of learning about animal health would be good for you (village meetings, classes, leaflets, radio broadcasts, internet information, etc)?)

Lack of educational environment

Education teaching in farms with face to face teaching.

**What changes could make your farm to more productive/ better?**

Need more green grass. I need equipment (machinery) to chop forage.

**What do you think is the future of this farm? (**Who will you pass your knowledge of farming on to?)

I want to increase the farm size with increased number of cows and increased cow size.

**Anything else you would like to tell us?**

“Welcome to our village. I hope that through education programme all the people of the village can gain good animal health knowledge.”

**7**

**Village**: **Date**: **Time**:

Interviewer: Andy Translator: Jadu

**Location/ who else is present / what else is happening?**

Home

**Name**: Farmer and mother **Male/ female:** **Age:** 42 and 65

**Your family and household**

**Who lives in your house?**

(Can we meet them please? What do they do? What do you want to do when grown up?)

Self:

Father

Mother

Wife: 40

Brother: 38

Sister in law: 35

Son: 16

Niece: 5

Grandson: 11 mths

**How is your family/ household supported?** (food, money, rent, etc).

Farming: paddy, mustard, veg for house

Daily labour (self, brother, sister in law.

**What animals does your family/ household have? Why are your animals important?** (Can we see your animals please? Why do you keep them? How do animals help to support your household? )

Cow: 1 (just bought)

Calf

Goat: 4

Duck

Chicken: 6

Ox: 2

**What is a ‘good’ animal?** How do you know?

(Cow and Ox): Soft hair, strong legs, long tail, hoof “tight shape, pointy, not spread”

Cow: Backside- strong, udder size, check history of cows mother- how much milk.

**How do you know if an animal is healthy?** How do you know if an animal is unhealthy?

Good mood, physical condition = healthy

Laying down, not cudding = unhealthy

**What things limit how much your animals give?** ( provide for your household?)

**Young Animals**

Where do you keep your calves? Can we see please?

**How many of your cows/ buffs/ goats had a baby in the last year?**

0

**Where are those baby animals now?**

**When a calf is born, can you teach me about the first day of its life?**

(When should it first drink milk (colostrum)? How much? What can you do to help it to be healthy? Who helps it?)

Daytime- cow licks calf clean

Night time- make a fire to warm calf and boil water

Wash udder w/ warm water

Get calf to drink ASAP. If calf stumbles when it starts to walk, steady it and take to teat. Can use fingers to encourage to suck and open mouth if required.

Take no “Pha-Ho”.

Give cow warm water to drink and placenta falls away quickly. (What do you do if the placenta doesn’t fall away quickly?) The placenta always falls.

Keep the navel clean to prevent flies.

**How much milk does an older calf drink and how often does it get it?** (Can you teach me how you do this? Who is responsible for this?)

[Mother takes over]

Take a little milk at 3 days, properly milk at 7 d- morning only.

Milk 4 teats and take 75% of milk.

After 15 – 30 days milk twice daily.

**Do your calves have any food apart from milk? Water?**( Where? How often? Who’s job?)

Rice water after 15 days. Grazing.

Shares cows feed: cooked food: rice polis, veg, banana stems and banana tree.

Every ones job, but mainly done by older people as young people working at labour.

**How much milk does your family get each day from your animals?** (What do you do with it? When do you first milk a cow/ buff /goat for drinking?)

Once daily milking- 2L daily

Twice daily milking 2.5L daily

**Adult Animals**

**Can you show me where your animals rest in the day? At night?**

Day- paddy, night- shed

**How do your animals get food? What**? (Who (family member) is responsible for this? Can we meet them please? How much land do you have? Can we see it?) **Where do your animals drink? How often?**

Rice straw- quantity given varies with availability- always give during flood, give at other times if plenty.

Cooked food in evening all year round

Salt water noon and evening.

Dhania stems also used as fodder.

**Did you vaccinate your animals last year? Why / why not?**

Yes. Cows and Ox for last 10 years. Protects from Soboka and other diseases.

Use because Forest Department initiatives provide for free.

**Do you de-worm animals? How? Why/ why not?**

If the Foresrt Department or some NGO provide, then we use. Tablets and injections for cows and ox. Sometimes I see small white (flat?) worms in dung sometimes. There is no local medicine for this.

**Do you do anything else to help your animals?**

**You live close to the forest reserve. Does this affect you?**

Crop raiding by elephant and boar, not usually rhino.

**Animal health education**

**Is there anyone who can treat your animals?** (What would you like the vet doctor to know more about?)

Yes. Kanholi Murray 2km and Sabzuri 5 km.

Dr is very good and knows a lot but I have to pay.

**Would you like to learn more about animal health? Why?**

Yes. I will treat my own animals and advise villagers.

**What problems are there for people trying to learn about animal health in your village?** ( Why? Which ways of learning about animal health would be good for you (village meetings, classes, leaflets, radio broadcasts, internet information, etc)?)

Lack of resources.

I am an older lady and may struggle to learn but the young people may struggle for a lack of time.

People are poor and go for daily labour so they lack the time to read.

Training with a good teacher. If a training course was provided it would be up to the individual to make the time or not.

**What changes could make your farm to more productive/ better?**

?

**What do you think is the future of this farm? (**Who will you pass your knowledge of farming on to?)

Sons and grandsons

**Anything else you would like to tell us?**

**8**

**Village**: **Date**: **Time**:

Interviewer: Andy Translator: Jadu

**Location/ who else is present / what else is happening?**

Middle street, home

**Name**: **~~Male~~/ female:** **Age:** 25

**Your family and household**

**Who lives in your house?**

(Can we meet them please? What do they do? What do you want to do when grown up?)

Self: Primary school teacher

Husband: 32, business, small grocery shop at house

**How is your family/ household supported?** (food, money, rent, etc).

Business

Farming: paddy, mustard

Teacher

**What animals does your family/ household have? Why are your animals important?** (Can we see your animals please? Why do you keep them? How do animals help to support your household? )

Cow: 2 sell calves, milk for house

Calf: 1

Goat: 4 sell

Duck

Chicken: 15 sell, eat, eggs, more chickens

Ox

Rent tractor when required

**What is a ‘good’ animal?** How do you know?

Good condition, eating well, good walking style. If these are poor then cow is weak or unhealthy

Husband buys cows, may go with friend

**How do you know if an animal is healthy?** How do you know if an animal is unhealthy?

**What things limit how much your animals give?** ( provide for your household?)

Tiger kills goats and cows. Calf killed last year. 2 goats also killed last year (in two months). These killings occurred during day time, near the river (at park boundary, used for grazing and watering animals), other villagers saw. This occurred over a period of 6 months.

**Young Animals**

Where do you keep your calves? Can we see please?

**How many of your cows/ buffs/ goats had a baby in the last year?**

2 cows – 2 calves

**Where are those baby animals now?**

1 living here

1 eaten by tiger

**When a calf is born, can you teach me about the first day of its life?**

(When should it first drink milk (colostrum)? How much? What can you do to help it to be healthy? Who helps it?)

Give cow cooked food- rice polis, veg, banana tree.

“Phay Who”(yellow milk)- take approx. 0.5L depend on cow size, drawn from all 4 teats

Cook phay- who (boil w/ sugar for 10- 15 mins until it boils down to make sweets). Give some sweets to cow and calf.

Calf drinks after milking is finished- approximately 1 – 1.5 hours old if he stands on own. Assist the calf to stand and suckle if required.

Nothing else is done.

First 7 – 10 days cow and calf together at all times.

**How much milk does an older calf drink and how often does it get it?** (Can you teach me how you do this? Who is responsible for this?)

Start milking cow at 7 – 10 days. Morning milking. Cow and calf separate at night- in the morning the calf drinks a little- then milk all 4 teats. Not milked empty- take 1 L / day.

Husband does.

**Do your calves have any food apart from milk? Water?**( Where? How often? Who’s job?)

No food until teeth grow (3 – 6 months).

Cooked meal- rice polis, veg, banana- same as mother

Water noon and evening.

Husband and wife do.

**How much milk does your family get each day from your animals?** (What do you do with it? When do you first milk a cow/ buff /goat for drinking?)

1 L / cow / day. Sell little only.

**Adult Animals**

**Can you show me where your animals rest in the day? At night?**

Shed night, paddy day

**How do your animals get food? What**? (Who (family member) is responsible for this? Can we meet them please? How much land do you have? Can we see it?) **Where do your animals drink? How often?**

Water at noon and evening

Cooked food in the evening

Husband does.

**Did you vaccinate your animals last year? Why / why not?**

Yes. Been doing for 7 years. Prevents Dz such as skin rash

**Do you de-worm animals? How? Why/ why not?**

Yes. Every 3 months. Injection or tablet. Never see worms in dung

**Do you do anything else to help your animals?**

**You live close to the forest reserve. Does this affect you?**

Crop raiding by elephants and wild boar. Only occasionally rhino.

Monkeys (Rhesus macaques) invade food store

**Animal health education**

**Is there anyone who can treat your animals?** (What would you like the vet doctor to know more about?)

Dr -5km (Sabjuri)

**Would you like to learn more about animal health? Why?**

Yes. Dr is not always available. I would like to learn more from doctors.

**What problems are there for people trying to learn about animal health in your village?** ( Why? Which ways of learning about animal health would be good for you (village meetings, classes, leaflets, radio broadcasts, internet information, etc)?)

“Training selected people would be more helpful. We lack the proper resources for learning here.”

“Teaching from someone with experience about animal health and farming would be the best way for people to learn and apply knowledge.”

(Reading materials?)” Yes, people can learn from them. A few might be interested, but not all. People here are too busy cultivating food.”

“The literacy rate here is about 30% (for what language? Assamese?). Yes, Assamese. A few people here can read Hindi, and English too. The young people read better, the old people less.”

**What changes could make your farm to more productive/ better?**

Don’t know

**What do you think is the future of this farm? (**Who will you pass your knowledge of farming on to?)

I would like mb son to have a good job

**Anything else you would like to tell us?**

“It is very good that you have come to us. This is a very good initiative. No one has ever come here before to do any kind of veterinary survey. Some people did a home survey, the school did its own survey and the census comes here. But no-one has been interested in our animals before.”

**9**

**Village**: **Date**: **Time**:

Interviewer: Andy Translator: Jadu

**Location/ who else is present / what else is happening?**

3 men, 2 ladies, 1 girl. Outside house, people working paddy in front. Man of this house too busy in paddy to interview, neighbour turned up so we are interviewing him first instead.

**Name**: **Male/ ~~female:~~** **Age:** 43

**Your family and household**

**Who lives in your house?**

(Can we meet them please? What do they do? What do you want to do when grown up?)

Wife

Son: 23 company job (MRL) temporary work in a refinery

Daughter: 19 study HS final

**How is your family/ household supported?** (food, money, rent, etc).

Farm; paddy, mustard, veg for home

**What animals does your family/ household have? Why are your animals important?** (Can we see your animals please? Why do you keep them? How do animals help to support your household? )

Cow 2 Sell milk, calves also

Calf 3 Sell sometimes at 4 – 5 yrs, males only, keep females for more cows

Goat 5 Selling, never eating

Duck

Chicken 4 Sell chickens, eat eggs

Ox

Rent tractor when required

**What is a ‘good’ animal?** How do you know?

(Another man arrives and joins in )

Height, face- especially teeth- age, legs, good hooves, muscle- particularly legs, strong backside.

When buying a cow I take an experienced guy with me to help

**How do you know if an animal is healthy?** How do you know if an animal is unhealthy?

Thin (bad sign), a good cow should have good muscle.

A cow should be in good mood and eat well. A sick cow stops eating or does less eating.

Healthy cow is active all day, a sick cow is less active and lays down.

**What things limit how much your animals give?** ( provide for your household?)

Not enough green grass available

**Young Animals**

Where do you keep your calves? Can we see please?

**How many of your cows/ buffs/ goats had a baby in the last year?**

2 cows, 3 calves- 1 each cow this year, 1 older calf (only one calf born last year)

**Where are those baby animals now?**

Here

**When a calf is born, can you teach me about the first day of its life?**

(When should it first drink milk (colostrum)? How much? What can you do to help it to be healthy? Who helps it?)

Take Pha-Hoo from cow, we take approximately half, about 1 litre, by milking all four teats half empty. The calf then drinks the other half of the Pha- Hoo. The calf drinks when it is about 30 minutes old.

**How much milk does an older calf drink and how often does it get it?** (Can you teach me how you do this? Who is responsible for this?)

Next milking at 10 – 12 days. Cow and calf together at all times until then.

After this cow and calf separated overnight. Release calf to go to cow, then catch calf again after a few minutes. Milk cow, then calf goes back to cow for the day.

Cow is milked once daily in morning.

My wife does this.

**Do your calves have any food apart from milk? Water?**( Where? How often? Who’s job?)

Start to eat about 1 month, start grazing. Calf should do this on it’s own. Cow goes grazing and the calf goes with her.

Taken water 3 times daily.

**How much milk does your family get each day from your animals?** (What do you do with it? When do you first milk a cow/ buff /goat for drinking?)

1.5 L per day (per cow?). Sell most, some for house.

**Adult Animals**

**Can you show me where your animals rest in the day? At night?**

Paddy day, shed night

**How do your animals get food? What**? (Who (family member) is responsible for this? Can we meet them please? How much land do you have? Can we see it?) **Where do your animals drink? How often?**

Evening rice straw and rice husk.

Water 3 times daily

My wife does this work.

**Did you vaccinate your animals last year? Why / why not?**

This year, yes. Usually we don’t use vaccine, I don’t really know why, we just don’t.

We only vaccinate when the government or some organisation (NGO) come to the village and do it.

**Do you de-worm animals? How? Why/ why not?**

Dr does it, only the calves with an oral medicine. Sometimes we see worms in the dung, we don’t do anything. We see them soon after treatment. Long flat worms- “Fita- pellu”

**Do you do anything else to help your animals?**

Clean shed

Wash cows every 3 – 6 months

**You live close to the forest reserve. Does this affect you?**

Crop raiding- elephants, boar, wild buffalo, (only occasionally rhino), parakeets.

**Animal health education**

**Is there anyone who can treat your animals?** (What would you like the vet doctor to know more about?)

Compounder in Sabzuri. Comes when needed.

**Would you like to learn more about animal health? Why?**

I would like tyo learn from a Dr or compounder how to treat my own animals.

**What problems are there for people trying to learn about animal health in your village?** ( Why? Which ways of learning about animal health would be good for you (village meetings, classes, leaflets, radio broadcasts, internet information, etc)?)

“Practical teaching. Meetings would be very useful. I could learn something from leaflets also.”

Insufficient Dr’s and resources. No one ever teaches us.

“Because it is for me I will have to manage time, and I will manage.”

**What changes could make your farm to more productive/ better?**

No changes.

“To produce more milk I would like to cross breed some of my animals.”

**What do you think is the future of this farm? (**Who will you pass your knowledge of farming on to?)

I don’t want my children to carry on the farm.

“In my younger age I did not receive things (education). For this reason I have provided for them education and other things. That is why I will be very happy if they get some jobs.”

**Anything else you would like to tell us?**

“If you provide us some training we can be very grateful.”

**10**

**Village**: **Date**: **Time**:

Interviewer: Andy Translator: Jadu

**Location/ who else is present / what else is happening?**

Home. Son, female friend and her daughter (approx. 9 yrs)

**Name**: **~~Male~~/ female:** **Age:** 33 years (initially forgot)

**Your family and household**

**Who lives in your house?**

(Can we meet them please? What do they do? What do you want to do when grown up?)

Self

Husband

Daughter 15 8 standard

Son 18 farming

**How is your family/ household supported?** (food, money, rent, etc).

Farming: paddy, mustard, Daal, veg- sell what family doesn’t eat

Daily labour

Weaving

**What animals does your family/ household have? Why are your animals important?** (Can we see your animals please? Why do you keep them? How do animals help to support your household? )

Cow 3 milk- sell and drink, calves

Calf 5 keep strong, sell weak

Goat 0

Duck 5 + 18 ducklings eat and sell ducks and eggs and make more ducks

Chicken 8-10 as above

Ox 2

**What is a ‘good’ animal?** How do you know?

Cow: height, strong body, any colour, udder size (big = more milk), eating well

Ox: Leg muscles, walking style, hump (big = good), big height, long tail (keep flies away)

Husband would buy new animal. He goes alone- very knowledgable

**How do you know if an animal is healthy?** How do you know if an animal is unhealthy?

Stop eating, mood change: bad mood, lethargic, lays down more

Touch animal- feel fever, hair stands up

**What things limit how much your animals give?** ( provide for your household?)

Tiger. Never killed one of my cattle. Killed the neighbours cattle.

Small jungle cats- eat ducks and chickens.

**Young Animals**

Where do you keep your calves? Can we see please?

**How many of your cows/ buffs/ goats had a baby in the last year?**

3 cows, 5 calves. All calved this year, 2 older calves here + 1 sold.

Cow died 2016 dysentery, calf died of no milk afterwards (was 7 -8 wks old)

**Where are those baby animals now?**

**When a calf is born, can you teach me about the first day of its life?**

(When should it first drink milk (colostrum)? How much? What can you do to help it to be healthy? Who helps it?)

Clean calf, give calf “Phao- Hoo” (15 – 20 mins) then milk phao hoo for house- 0.5L (udder not empty)

Help calf to stand and drink. Open mouth and put on teat. “It can be very difficult to feed new born calves.”

“If it drinks the first milk it will become strong and healthy. If it does not drink it will become thin and weak. It may die if it does not get the yellow milk.”

**How much milk does an older calf drink and how often does it get it?** (Can you teach me how you do this? Who is responsible for this?)

Don’t milk cow for 30 days, cow and calf together all the time for 30 days.

After 30 days- separate at night, after midnight. Milk cow at 7am, don’t milk empty- get 1.5 – 2 L

**Do your calves have any food apart from milk? Water?**( Where? How often? Who’s job?)

Grazing only, start to eat a little at 3 months.

Water 3 times daily

Wife responsibility.

**How much milk does your family get each day from your animals?** (What do you do with it? When do you first milk a cow/ buff /goat for drinking?)

1.5 – 2 L cow/ day. House and sale.

**Adult Animals**

**Can you show me where your animals rest in the day? At night?**

Paddy, shed

**How do your animals get food? What**? (Who (family member) is responsible for this? Can we meet them please? How much land do you have? Can we see it?) **Where do your animals drink? How often?**

Grazing

Rice straw- all year

Rice polis (uncooked) in water

Always salt in water

Water 3 x daily

**Did you vaccinate your animals last year? Why / why not?**

Yes. Many years, only do if Forest Dept or some organisation (NGO) do.

Prevents dz: Kurra patta, pet phulla, dysentery.

**Do you de-worm animals? How? Why/ why not?**

Tablets to calf at 6 m/o and 12 m/o, from Dr or pharmacy.

Yes we see worms (“Pelu”), small and flat

**Do you do anything else to help your animals?**

Clean shed daily

Smoke shed to prevent flies and mosquitos in summer.

**You live close to the forest reserve. Does this affect you?**

Crop raiding: elephant, boar. Occasionally elephants damage houses and raid grain stores.

**Animal health education**

**Is there anyone who can treat your animals?** (What would you like the vet doctor to know more about?)

Dr knows a lot.

**Would you like to learn more about animal health? Why?**

Yes. I can look after my own animals and help my neighbours.

**What problems are there for people trying to learn about animal health in your village?** ( Why? Which ways of learning about animal health would be good for you (village meetings, classes, leaflets, radio broadcasts, internet information, etc)?)

“If you teach us we can learn. Practical training by some experts would be very helpful to us. But I can learn from posters leaflets and flags too.”

“We don’t have the environment for learning here. At my younger age I didn’t want to learn about these things. Now I want to learn because if I learn I can keep my own animals healthy and more productive,”

Only 10% can read here (guess)

“Many people in this village are farmers and raise cattle and don’t go for study and those kind of things. Only younger generation is interested in study and college.”

**What changes could make your farm to more productive/ better?**

**What do you think is the future of this farm? (**Who will you pass your knowledge of farming on to?)

Children will carry on working farm

**Anything else you would like to tell us?**

**11**

**Village**: **Date**: **Time**:

Interviewer: Andy Translator: Jadu

**Location/ who else is present / what else is happening?**

Outside house, wife, baby (belogs to another lady), son comes later

**Name**: **Male/ ~~female:~~** **Age:** 58

**Your family and household**

**Who lives in your house?**

(Can we meet them please? What do they do? What do you want to do when grown up?)

Self

Wife

4 sons 30, 27, 25 carpenters and farming, 22 farming

1 daughter in law 25

1 grand daughter 4

**How is your family/ household supported?** (food, money, rent, etc).

Farming: Paddy, mustard, black daal, sell vegetables: cabbage, brinjal, bottle gourd

“Mahori” village representative on Forest Dept labour committee- oversees work- receives same money as labourers.

**What animals does your family/ household have? Why are your animals important?** (Can we see your animals please? Why do you keep them? How do animals help to support your household? )

Cow 1 milk and dung 1L sale, feed calf and sale

Calf 2 sale

Goat 4 sale

Duck

Chicken

Ox

Cultivate: rent tractor

Ox killed by tiger 10 yrs ago, at that time tiger killed 13 cattle in 2 mths.

Want to get more ox, ox makes dung for compost, don’t have to pay tractor. Can’t afford outlay cost for ox (“money problem”).

**What is a ‘good’ animal?** How do you know?

Legs, walking style, health, size

“I don’t have that knowledge”

“I only look at its legs and its walking style and I guess if it is a good animal.”

“I take an experienced man from the village when I go to buy cattle and I listen to what he says.”

**How do you know if an animal is healthy?** How do you know if an animal is unhealthy?

Bad mood, dripping saliva, ears down, difficulty breathing

**What things limit how much your animals give?** ( provide for your household?)

Less availability of grass.

Unable to buy good quality foods for animals. We don’t have that kind of money so we are unable to provide good nutrition. Sometimes I buy food, but not enough for all animals and this reduces productivity. Husk and grain that I mix in cooked food.

Animal nutrition is biggest problem from March to July.

“March to April (before flood) is the best time to vaccinate animals. Private vaccine is better than Govt. If vaccination is done in proper time saboka never occurs. Vaccine also for Gol phulla, Pet phulla and Go Basanta. Used to consult Dr, but will always vaccinate if vaccine available, but because of poor availability of vaccine Forest Dept don’t always come.

**Young Animals**

Where do you keep your calves? Can we see please?

**How many of your cows/ buffs/ goats had a baby in the last year?**

1 cow, 1 calf (2016), 1 older calf (2015)

**Where are those baby animals now?**

Here

**When a calf is born, can you teach me about the first day of its life?**

(When should it first drink milk (colostrum)? How much? What can you do to help it to be healthy? Who helps it?)

If dystocia- farmer correct- most people know this “All people in this village know about those things because the veterinary doctor is not always available.

Last delivered a calf here in 2007/8. “My wife did it, she knows a lot more about these things than me.”

Clean calf (womans job), clean face and nose, sometimes blow air in nostrils. Take calf out of shed to a sunny place if born in the day. Stands after 1.5 – 2 hours, take to cow and help to feed “Open mouth and put to teat, gently rub it’s head and then it starts to suck on its own. Sometimes you put your own finger in it’s mouth to encourage it to suck.

Calf drinks first, then we take some Phae Ho. Calf drinks for 2- 3 minutes. Take about 1 L depending on cow size, big cow, take more. After another 1 – 1.5 hour allow the calf to drink again. “Someone looks after the calf for the first 12 hours to check that it is drinking” (wife says). Calf is full in 2 minutes.

Next milking 10 days. Calf and calf are together all that time. Once milking, separate the calf from the mother at 1 or 2 o’clock – someone has to wake up. At 8 am milk cow- let calf out to drink first – 10 minutes – then milk cow- 1 -1.5L. The cow that died used to give 2.5L

**How much milk does an older calf drink and how often does it get it?** (Can you teach me how you do this? Who is responsible for this?)

Wife is in charge of calf and milking things.

**Do your calves have any food apart from milk? Water?**( Where? How often? Who’s job?)

Give nothing while still drinking milk. Starts to graze 5 – 6 months.

Water 1x or 2x daily, noon and evening, wife does. “Differs from house to house but 80 – 90% of women do this work. Tie cows in the paddy or shed is usually a man’s job. All other things are done by women. Man usually is mostly engaged with cultivation.”

“Man work is mostly hard work like cultivation, fetching heavy things or if the house is broken. Things women can’t do.” (Hard work or heavy work?) “Hard work.”

**How much milk does your family get each day from your animals?** (What do you do with it? When do you first milk a cow/ buff /goat for drinking?)

Sell 1 L daily.

0.5L for children and tea, this is sufficient. We have stopped drinking milk tea because it (milk?) gives older people gas problem.

**Adult Animals**

**Can you show me where your animals rest in the day? At night?**

Paddy day, shed night

**How do your animals get food? What**? (Who (family member) is responsible for this? Can we meet them please? How much land do you have? Can we see it?) **Where do your animals drink? How often?**

Rice straw (whole year) and cooked food- rice husk, vegetables and vegetable leaves.

During flood time cooked food includes chopped banana tree.

Water 2x daily, noon and evening.

Man and woman’s job, whoever is available.

**Did you vaccinate your animals last year? Why / why not?**

Yes. Since 2001. Prevents Dz.

Pet-phulla, Gol-phulla, Saboka, Bo-santa: vaccine different for different diseases.

“I always keep in touch with Forest Dept and use vaccine if available. If vaccine is not available sometimes we go to the pharmacy and buy it privately and do it ourselves.”

**Do you de-worm animals? How? Why/ why not?**

Yes, tablets- not frequently- at 1 year and after 2 or 3 years. Goats and calves. “Usually goats aren’t affected, it is mostly the calves. (Do you see worms in dung?) Son says: yes I have seen in the village (please describe) fat white worms in calf dung (show photo of T vit in dung) yes, like that.

**Do you do anything else to help your animals?**

**You live close to the forest reserve. Does this affect you?**

Tiger killing cows

Crop raiding and destruction of houses by elephant. Rhino, deer and wild boar also raid crops.

**Animal health education**

**Is there anyone who can treat your animals?** (What would you like the vet doctor to know more about?)

“He comes when we call. I can’t say he is a good doctor. He is good for small problems but if the case is major sometimes the animals die.”

“It would be good for us if he knew a lot.”

**Would you like to learn more about animal health? Why?**

“Yes. Doctor takes some fees. If I know these kind of things I would not have to give money to anyone. I could buy tablets and treat myself.”

**What problems are there for people trying to learn about animal health in your village?** ( Why? Which ways of learning about animal health would be good for you (village meetings, classes, leaflets, radio broadcasts, internet information, etc)?)

“Because I am growing old day by day, theory will not be enough for me. I need practical training. But for my sons, they can learn. They will benefit from theory and practical training.”

**What changes could make your farm to more productive/ better?**

Because of flood, this ground is not high enough. I want to buy improved quality cows but because of flood problem I am not buying. I want to make this ground higher, then perhaps I will buy.”

I want to make a good shed for the cows and then I can buy good quality cows and make more milk. I want to make a good shed with some metals and then I can protect them from the tiger.

**What do you think is the future of this farm? (**Who will you pass your knowledge of farming on to?)

“I am teaching my sons and they will carry on farming here. I am teaching all of my sons to be independent by farming, carpentering, and any kind of thing that they can do to make their lives better.”

“I am doing village politics but I don’t want my sons to become politicians.”

**Anything else you would like to tell us?**

“It is good that you are writing down everything that I am saying. You are actually listening to what I am saying.”

“I am very grateful that you have come here. We are hoping to get more from you than this survey. Not in a physical manner but teaching us so we can help ourselves. We are poor people raising cows and ox. Our biggest problem here is the tiger. Tiger causes a lot of damage. If it kills a cow or an ox worth Rs 30,000 how can we deal with that. We need help from Forest Dept or some NGOs to deal with this problem.”

“At first I went into politics unwillingly because a Party Member (Congress) came to this village and said that we need someone to represent your village so I went with it to represent my village. Now I release that it’s not worth it. I am not getting anything from this politics thing. I have to spend my own money every time I go somewhere, they give me nothing.”

**12**

**Village**: **Date**: **Time**: Interviewer: Andy Translator: Jadu

**Location/ who else is present / what else is happening?**

Home. Father and a lad present

**Name**: **Male/ ~~female:~~** **Age:** 35

**Your family and household**

**Who lives in your house?**

(Can we meet them please? What do they do? What do you want to do when grown up?)

Self: carpenter

Mother

Father

Brother: 30, carpenter

Brother 26

Wife

Sister in law

Daughter: 6

Son: 1.5

Niece: 4

**How is your family/ household supported?** (food, money, rent, etc).

Farming: paddy, mustard, vegetables- home only

Carpenter

**What animals does your family/ household have? Why are your animals important?** (Can we see your animals please? Why do you keep them? How do animals help to support your household? )

Cow: 1. Milk and calves

Calf: 2 (1 older)- sell at 4 years.

Goat : 10. Sell, never eat, but we would if we wanted,

Duck : 10. Eat, never sell. Eggs- some for eating, some for more ducks.

Pigeon: 6. Eat never sell

Chicken

Ox: 2

**What is a ‘good’ animal?** How do you know?

Ox: Legs- muscle, tells you their strength. Long legs will grow more big size.

Tail- long keep away flies and mosquitos

Cow: Fat (healthy) belly, height- but body size more important. Big udder.

**How do you know if an animal is healthy?** How do you know if an animal is unhealthy?

Mood- low mood if sick, ears drooped. Belly pain- looks round.

**What things limit how much your animals give?** ( provide for your household?)

Availability of grass

After flood- no grass in this area.

**Young Animals**

Where do you keep your calves? Can we see please?

**How many of your cows/ buffs/ goats had a baby in the last year?**

1 cow, 1 small calf, 1 large calf

**Where are those baby animals now?**

Here

**When a calf is born, can you teach me about the first day of its life?**

(When should it first drink milk (colostrum)? How much? What can you do to help it to be healthy? Who helps it?)

Call Dr if dystocia

Look after calf, if he can’t stand or suck, help him. If unable to drink- feed with bottle (100 – 200ml) if not fed in 2 hours.

Milk out 1^st^ Phae- Ho as soon as cow stands up. Don’t milk empty, if 2 L cow- take 1L Phae - Ho.

Give cow food including pepper- boiled veg to strengthen cow.

**How much milk does an older calf drink and how often does it get it?** (Can you teach me how you do this? Who is responsible for this?)

Next milking at 3 days – throw milk in river, and at 5 days throw in river. People avoid stepping on milk. My father does this, it is religion (seems unhappy about this). Repeat every 2 days until 11 days. Milk once daily until calf is grazing, then milk twice daily.

Increase separation time gradually, separate at 3 am, 2 am, 1 am.

At 8 am- calf drinks for 2- 3 mins- then milk 1.5L- rest for calf.

**Do your calves have any food apart from milk? Water?**( Where? How often? Who’s job?)

Starts to graze at 3 m/o

If belly goes fat (between 4 – 12 mths old) – know worms present. Give tablets.

Seen fat worms.

**How much milk does your family get each day from your animals?** (What do you do with it? When do you first milk a cow/ buff /goat for drinking?)

Once daily: 1 – 1.5L daily – all for house, sell none.

Twice daily: 2.5L daily

Father does.

**Adult Animals**

**Can you show me where your animals rest in the day? At night?**

Graze paddy- tied, shed at night.

**How do your animals get food? What**? (Who (family member) is responsible for this? Can we meet them please? How much land do you have? Can we see it?) **Where do your animals drink? How often?**

Cook food- vegetables, wheat grains, rice polis

Jaggery after giving birth. Father does.

Water: noon and evening- father does.

**Did you vaccinate your animals last year? Why / why not?**

Yes. 10 years. Prevents Dz spreading in national park. Stop animals going sick- Kura- phatta, Pet- phulla, Leg Dz (not sure what Dz’s). Forest Dept did last year.

**Do you de-worm animals? How? Why/ why not?**

Calves 4 – 12 mths if belly big.

**Do you do anything else to help your animals?**

Give food at proper time.

Change grazing position.

**You live close to the forest reserve. Does this affect you?**

Crop raiding and damage done by wild animals: elephant, rhino wild boar, deer.

There are no positive things about having the park here.

**Animal health education**

**Is there anyone who can treat your animals?** (What would you like the vet doctor to know more about?)

Very good Dr, but sometimes cannot save 1 or 2 animals. 3km away. Have to pay according to medicines provided- affordable.

**Would you like to learn more about animal health? Why?**

“Yes, so I can treat my own animals and teach other people in the village also.”

**What problems are there for people trying to learn about animal health in your village?** ( Why? Which ways of learning about animal health would be good for you (village meetings, classes, leaflets, radio broadcasts, internet information, etc)?)

I can only learn by practical teaching. I can read, but I can’t learn by reading a book.

Posters and visual learning (asked about this), some difficulties may arise.

Yes, I can manage time to learn.

**What changes could make your farm to more productive/ better?**

Make proper, clean shed for animals.

Make it clean to prevent sickness.

**What do you think is the future of this farm? (**Who will you pass your knowledge of farming on to?)

I want to make this farm bigger, but I cannoy because of my economic condition.

“I want my children to have good jobs, not farming.”

**Anything else you would like to tell us?**

“No. I have nothing else to say.”

**13**

**Village**: **Date**: **Time**:

Interviewer: Andy Translator: Jadu

**Location/ who else is present / what else is happening?**

Inside house, old man and small son also present, then brother arrives and joins interview. Vegetarian family.

**Name**: Two brothers **Male~~/ female:~~** **Age:** 52

**Your family and household**

**Who lives in your house?**

(Can we meet them please? What do they do? What do you want to do when grown up?)

“I learned about raising cows from my father. Most people in this village do not know about raising cows.”

Self

Wife

Son 10

Grandson 6

(Has 4 daughters, married, living other places)

I wants my son to be well educated and have a job. I am also teaching him morals and manners. He is getting good marks in school and his teachers are looking after him.

**How is your family/ household supported?** (food, money, rent, etc).

Farming: paddy, dhania, daal, (previously mustard, went on daal cultivation training course)

Carpenter (for 30 years)

**What animals does your family/ household have? Why are your animals important?** (Can we see your animals please? Why do you keep them? How do animals help to support your household? )

Cow: 1 gives milk and calves

Calf : 3 keep best ones and sell others. Haven’t sold one for 15 years. (how does this add up?)

Goat : 2 Gave to other people to raise (“Adhi”) 50/50 split profit.

Duck

Chicken

Ox: 0 Prevoiusly had 2 but sold as insufficient grass

Now I use rent tractor. I will but one ox when my older calf is big enough to use in the field. I prefer to work with ox than tractor. (Why?) The production is better when you use ox compared with tractor. ((Why do you think that is?) I don’t know why.

I sell my ox when they are old, or if they have some problem, like fever. I sold a cow that was not getting pregnant.

**What is a ‘good’ animal?** How do you know?

Cow: short height, fat round belly, soft hair, no horns.

Ox: Big face, medium size horn, strong front side, long tail, round front (when viewed from front)

Prefer red or white animals, but this is secondary consideration.

**How do you know if an animal is healthy?** How do you know if an animal is unhealthy?

Mood. Sick cow is sad, healthy cow is energetic and in good mood. Sick cow is laying down, laying down is not a healthy sign, it has less activity and does less eating and drinking.

**What things limit how much your animals give?** ( provide for your household?)

**Young Animals**

Where do you keep your calves? Can we see please?

**How many of your cows/ buffs/ goats had a baby in the last year?**

1 cow, 1 calf

**Where are those baby animals now?**

Alive, here.

**When a calf is born, can you teach me about the first day of its life?**

(When should it first drink milk (colostrum)? How much? What can you do to help it to be healthy? Who helps it?)

If dystocia occurs- fetch …..’s wife. Usually it is women who help animals to be born, they understand this work.

Pull legs and tail (makes legs strong), Blow air in ear, eye, nose and mouth (clears blockages), male calf- press hump (makes bigger).

Protect calf from other animals.

If winter make fire to keep warm

Clean calf with warm water; give cow hot water to drink.

Give cow bamboo leaf and sugar cane leaf to eat- placenta falls quickly.

Tie rice straw through calf’s mouth after pulling legs, leave for 10 minutes.

When calf tries to stand- help it to feed from mother.

Phay Hoo- calf drinks 15 -20 minutes then milk cow for Phay Hoo for house- 0.5L, sometimes less. Estimate amount of milk from the size of the cow- half for the calf.

Don’t milk cow for 7 days, cow and calf together the whole time.

**How much milk does an older calf drink and how often does it get it?** (Can you teach me how you do this? Who is responsible for this?)

After 7 days, separate cow and calf at midnight and increase this time over some days. In morning take cow out and tie in a clean place, untie calf- let down occurs- allow calf to suck for 2 minutes then remove calf- milk cow- take half milk, leave the rest for the calf.

Calf must drink as much as possible, otherwise becomes weak.

**Do your calves have any food apart from milk? Water?**( Where? How often? Who’s job?)

After 6-7 months: rice husk and rice polis in water with salt

Water 3 times daily.

**How much milk does your family get each day from your animals?** (What do you do with it? When do you first milk a cow/ buff /goat for drinking?)

1.5L / cow / day. Sell 1L.

Wife does milk and calf jobs.

**Adult Animals**

**Can you show me where your animals rest in the day? At night?**

Day: tie in paddy, Night: shed

**How do your animals get food? What**? (Who (family member) is responsible for this? Can we meet them please? How much land do you have? Can we see it?) **Where do your animals drink? How often?**

Left over veg and veg leaves, rice husk, rice polis, grain polis with water and salt

Water 3 times daily

Husband and wife both do

Flood time: chopped banana tree with rice husk and rice polis and rice straw.

**Did you vaccinate your animals last year? Why / why not?**

Yes, last 10 years.

Forest Dept tell us vaccination can get rid of Saboka and prevent spread to wild animals.

Also Basanta and Bohonta.

**Do you de-worm animals? How? Why/ why not?**

Yes, tablets from Dr, cow and calf and goats treated.

**Do you do anything else to help your animals?**

Vitamin tablets sometimes.

**You live close to the forest reserve. Does this affect you?**

I am very positive about the forest. There are many varieties of wild animals and many people come to see them. Many people are engaged in this work. We are very lucky because the rhino is the pride of Assam.

“I have no problems with the wildlife. God made all animals equal and all have a right to live. If sometimes animals venture out of the park and damage crops we receive compensation. It is not a big deal for me.”

“People have become very materialistic in this time. They are not loving the wild animals like they did in ancient times. If we can teach people to love all animals it make a really big difference, and to me also, it will make me very happy.”

**Animal health education**

**Is there anyone who can treat your animals?** (What would you like the vet doctor to know more about?)

Dr in Sabzuri 4km away and in Kanduli Muri 1.5 Km away. Good Drs and they come when they are called.

**Would you like to learn more about animal health? Why?**

I want to learn to treat animals and to raise in a better manner.

**What problems are there for people trying to learn about animal health in your village?** ( Why? Which ways of learning about animal health would be good for you (village meetings, classes, leaflets, radio broadcasts, internet information, etc)?)

Can read books but some things can be learned only from practical training. Posters and leaflets are also helpful

Lack of resources, the flood. I have to work hard to send my boys to school so time is limited.

**What changes could make your farm to more productive/ better?**

**What do you think is the future of this farm? (**Who will you pass your knowledge of farming on to?)

I want to make farm bigger, buy jerseys with more milk production and get high quality goats.

Food will be available for this but we will have to prepare well for the flood.

“I think my son will carry on farming. He likes to copy me and work the farm and he asks many questions and in anxious to learn.”

**Anything else you would like to tell us?**

**14**

**Village**: **Date**: **Time**: Interviewer: Andy Translator: Jadu

**Location/ who else is present / what else is happening?**

Outside home, forest staff friend (30), niece, nephew

**Name**: **~~Male~~/ female:** **Age:** 34

**Your family and household**

**Who lives in your house?**

(Can we meet them please? What do they do? What do you want to do when grown up?)

Self

Mother

Brother 40

Sister in law 35

Nephew 20 (HS 1^st^ year)

Niece 18 (HS final year)

**How is your family/ household supported?** (food, money, rent, etc).

Farming: paddy, mustard, veg (sell and house)

Weaving

Occasional labour

**What animals does your family/ household have? Why are your animals important?** (Can we see your animals please? Why do you keep them? How do animals help to support your household? )

Cow: 2 calves and milk

Calf: 2 keep females to increase number, sell male at 3-4 years- sell for money problem or tiger problem.

Goat: 9 sell, occ eat (Bihu, Pooja)

Duck: 4 (Haa) selling

Chicken: 20 eating and selling and making more chickens (mostly eating)

Ox: 2 (Halua) Use from 3- 4 years, keep for (or until?) 7 -8 years then sell. If good healthy male calves then train themselves, otherwise buy. Training ox is hard, brother does with help from experienced men in village.

**What is a ‘good’ animal?** How do you know?

Healthy.

Cow: Length and height of cow, soft hair, round belly.

Ox: I don’t know much

**How do you know if an animal is healthy?** How do you know if an animal is unhealthy?

Stop grazing, laying down. By seeing I just know.

**What things limit how much your animals give?** ( provide for your household?)

**Young Animals**

Where do you keep your calves? Can we see please?

**How many of your cows/ buffs/ goats had a baby in the last year?**

2 cows, 2 calves

**Where are those baby animals now?**

**When a calf is born, can you teach me about the first day of its life?**

(When should it first drink milk (colostrum)? How much? What can you do to help it to be healthy? Who helps it?)

If dystocia- call Dr., no experienced men in this area.

Clean cow, clean calf, clean navel with coconut oil, watch that no birds peck at navel cord.

Feed cow- cabbage or any available veg, rice husk.

Observe carefully and check that calf is drinking.

Phaa Hoo: before calf drinks milk out 1L from cow using all 4 teats. Make Phaa Hoo sweets, make also a lucky string of Phaa Hoo beads and put round cow and calf neck. Calf drinks half after cow is milked. Cow is milked 1 hour after calf is born, calf first drinks at 1.5 hours old.

Concerned about diarrhoea so prevent calf from drinking “Not drinking first yellow milk prevents calf from having diarrhoea.”

**How much milk does an older calf drink and how often does it get it?** (Can you teach me how you do this? Who is responsible for this?)

Next milking after 10 days, calf is fully drinking for 10 days, cow and calf together, cow is grazed but not tied when she has young calf.

Milked once daily for first month, then twice daily. Separate cow and calf at 2am, in morning let calf drink for 3 minutes then milk cow for 1 – 1.5L, some milk left in udder for calf.

**Do your calves have any food apart from milk? Water?**( Where? How often? Who’s job?)

Start grazing at 1.5 months old.

Give water at noon and evening.

Self or mother does.

**How much milk does your family get each day from your animals?** (What do you do with it? When do you first milk a cow/ buff /goat for drinking?)

1 - 1.5L cow/day, sell at little

If milking twice daily- nearly 2L

I milk the cow

**Adult Animals**

**Can you show me where your animals rest in the day? At night?**

Shed night, paddy day

**How do your animals get food? What**? (Who (family member) is responsible for this? Can we meet them please? How much land do you have? Can we see it?) **Where do your animals drink? How often?**

Cooked food- veg, rice water, rice husk, salt

During flood- banana tree chopped, elephant grass, rice straw, weeds from wetland.

Water 3- 4 times daily in flood, other times twice daily.

Self and mother do.

**Did you vaccinate your animals last year? Why / why not?**

Yes. 10 years. Prevent disease- Hagoni, Kura patta, Pet fulla.

**Do you de-worm animals? How? Why/ why not?**

No.

“Pel- lou”: worms

“Nee- mok”: salt

**Do you do anything else to help your animals?**

Concentrate mixture (powder)- give to lactating cows to produce more milk. Cooked like rice husk, smells slightly like yeast. 200- 300g mixed with veg or rice husk once daily.

**You live close to the forest reserve. Does this affect you?**

Crop raiding- wild animals, elephant, rhino

Tiger killing cattle. 4 animals- 3 cows and 1 calf (3 year old). Last time May 2014, occurred in shed- saw tiger and scared tiger away. WWF/ Forest Dept compensation Rs4000 (WWF Rs2500, FD Rs1500) per cow. Took 6 months to pay, Rs20,000 total. Bought new cow.

New cow Rs 15k, Ox Rs 30K – 40K/ pair or >15K single.

**Animal health education**

**Is there anyone who can treat your animals?** (What would you like the vet doctor to know more about?)

Dr 5km away, comes when we call if he is available. He is a good Dr.

**Would you like to learn more about animal health? Why?**

“Yes. If I could do primary care (first aid?) by myself and then I wouldn’t have to wait until the Dr comes.”

**What problems are there for people trying to learn about animal health in your village?** ( Why? Which ways of learning about animal health would be good for you (village meetings, classes, leaflets, radio broadcasts, internet information, etc)?)

“I can learn from books, but I also have to learn practically. Actually, in our place there is no government training and we don’t have any resources,”

“Yes, I can manage time to learn, but it will be difficult for me in flood season.”

**What changes could make your farm to more productive/ better?**

“If I got good blood (breeding) animals then my production will be more. I want to increase my number of cows and goats.”

**What do you think is the future of this farm? (**Who will you pass your knowledge of farming on to?)

“I will continue and then the next generation will take over. It continues from generation to generation. We have always lived here.”

**Anything else you would like to tell us?**

“If training can be provided it will make a difference to us.”

Nose roping: “ Tie the animals legs together and push it over, grab it’s head and piece nose. The spike pulls the rope through. We use a bamboo spike. This boy has done it. Pushing down a bull is hard, it takes three or four people to do it.”

Castration: “We get the Dr to attend the bullock. He uses a clamp (burdizo) and gives injection. We don’t use the village people for this.”

**14**

**Village**: DFP **Date**: **Time**:

Interviewer: Andy Translator: Jadu

**Location/ who else is present / what else is happening?**

At home with husband

**Name**: **~~Male~~/ female:** **Age:** Thinks for a long time. “Maybe 45? I have forgotten.”

**Your family and household**

**Who lives in your house?**

(Can we meet them please? What do they do? What do you want to do when grown up?)

Self

Husband

Son 30 Carpenter

Son 27 Carpenter

Daughter in law 27 Shop

Daughter in law 26

Grandson 5 I want them to have good jobs like doctor. Boy says policeman

Grandson 4.5

**How is your family/ household supported?** (food, money, rent, etc).

Farming: paddy, mustard, veges- mostly for eating, occasionally sell

Shop

Carpentering

**What animals does your family/ household have? Why are your animals important?** (Can we see your animals please? Why do you keep them? How do animals help to support your household? )

Cow: 2 calves and milk- for house and sale

Calf: 2 keep females if we can, males always sold. We sell especially if we need money. It is like a cow bank

Goat: 0

Duck: 7 (Haa) eat and eggs for selling, eating and more ducks

Chicken: don’t keep now as we have ducks

Ox: 2 (Halua) Cultivation. Buy or use our own. My husband trains the ox.If he buys an ox he goes alone, he is very knowledgable. Buy the ox when it has two big (adult) teeth, start work (or sell on ox calf) when it has 6 adult teeth. Sell ox when it is about 10 years old. We guess the age by looking.

**What is a ‘good’ animal?** How do you know?

Cow: Legs should be long, belly round, long tail and big udder. Cow with big belly can eat more and will give birth to a strong calf.

Ox: Strong legs, long legs, good hooves- perfect shape- neat and tight. Horns should go up not out.

**How do you know if an animal is healthy?** How do you know if an animal is unhealthy?

Physique, dung- watery or very smelly, stops eating, change of behaviour, disease.

**What things limit how much your animals give?** ( provide for your household?)

**Young Animals**

Where do you keep your calves? Can we see please?

**How many of your cows/ buffs/ goats had a baby in the last year?**

2 cows, 2 calves

**Where are those baby animals now?**

**When a calf is born, can you teach me about the first day of its life?**

(When should it first drink milk (colostrum)? How much? What can you do to help it to be healthy? Who helps it?)

If dystocia- call Dr., no experienced men in this area.

Clean cow, clean calf, clean navel with coconut oil, watch that no birds peck at navel cord.

Feed cow- cabbage or any available veg, rice husk.

Observe carefully and check that calf is drinking.

Phaa Hoo: before calf drinks milk out 1L from cow using all 4 teats. Make Phaa Hoo sweets, make also a lucky string of Phaa Hoo beads and put round cow and calf neck. Calf drinks half after cow is milked. Cow is milked 1 hour after calf is born, calf first drinks at 1.5 hours old.

Concerned about diarrhoea so prevent calf from drinking “Not drinking first yellow milk prevents calf from having diarrhoea.”

**How much milk does an older calf drink and how often does it get it?** (Can you teach me how you do this? Who is responsible for this?)

Next milking after 10 days, calf is fully drinking for 10 days, cow and calf together, cow is grazed but not tied when she has young calf.

Milked once daily for first month, then twice daily. Separate cow and calf at 2am, in morning let calf drink for 3 minutes then milk cow for 1 – 1.5L, some milk left in udder for calf.

**Do your calves have any food apart from milk? Water?**( Where? How often? Who’s job?)

Start grazing at 1.5 months old.

Give water at noon and evening.

Self or mother does.

**How much milk does your family get each day from your animals?** (What do you do with it? When do you first milk a cow/ buff /goat for drinking?)

1 - 1.5L cow/day, sell at little

If milking twice daily- nearly 2L

I milk the cow

**Adult Animals**

**Can you show me where your animals rest in the day? At night?**

Shed night, paddy day

**How do your animals get food? What**? (Who (family member) is responsible for this? Can we meet them please? How much land do you have? Can we see it?) **Where do your animals drink? How often?**

Cooked food- veg, rice water, rice husk, salt

During flood- banana tree chopped, elephant grass, rice straw, weeds from wetland.

Water 3- 4 times daily in flood, other times twice daily.

Self and mother do.

**Did you vaccinate your animals last year? Why / why not?**

Yes. 10 years. Prevent disease- Hagoni, Kura patta, Pet fulla.

**Do you de-worm animals? How? Why/ why not?**

No.

**Do you do anything else to help your animals?**

Concentrate mixture (powder)- give to lactating cows to produce more milk. Cooked like rice husk, smells slightly like yeast. 200- 300g mixed with veg or rice husk once daily.

**You live close to the forest reserve. Does this affect you?**

Crop raiding- wild animals, elephant, rhino

Tiger killing cattle. 4 animals- 3 cows and 1 calf (3 year old). Last time May 2014, occurred in shed- saw tiger and scared tiger away. WWF/ Forest Dept compensation Rs4000 (WWF Rs2500, FD Rs1500) per cow. Took 6 months to pay, Rs20,000 total. Bought new cow.

New cow Rs 15k, Ox Rs 30K – 40K/ pair or >15K single.

**Animal health education**

**Is there anyone who can treat your animals?** (What would you like the vet doctor to know more about?)

**Would you like to learn more about animal health? Why?**

**What problems are there for people trying to learn about animal health in your village?** ( Why? Which ways of learning about animal health would be good for you (village meetings, classes, leaflets, radio broadcasts, internet information, etc)?)

**What changes could make your farm to more productive/ better?**

**What do you think is the future of this farm? (**Who will you pass your knowledge of farming on to?)

**Anything else you would like to tell us?**

**16 and 17**

**Village**: **Date**: **Time**: Interviewer: Andy Translator: Jadu

**Location/ who else is present / what else is happening?**

**Name**: (Husband) **Male/ female:** **Age:** 44

(wife) 38

(Friend) 55

**Your family and household**

**Who lives in your house?**

(Can we meet them please? What do they do? What do you want to do when grown up?)

**16**

Selfs

Daughter 18 BSc botany 2^nd^ semester

Son 16 HS 1^st^ year

Hopes children will get jobs- depends on what qualifications they get and what they want to do- never thought about what would happen if children did not want to farm later on

**17**

Wife

Son 23 Works for a company in Hyderabad- feels proud but is sad that he is not here

Daughter 28 Helps in house

**How is your family/ household supported?** (food, money, rent, etc).

**16**

Farming: Paddy, mustard, veg- toms, dhania, carrots, all kinds- sell veg in Bokahat

Business: sell (and transport on motor bike) supplies e.g. bags, candles in Bokahat

**17**

Farming: Paddy, mustard, veges- all kinds- eat and sell

**What animals does your family/ household have? Why are your animals important?** (Can we see your animals please? Why do you keep them? How do animals help to support your household? )

**16**

Cow: 1 milk

Calf: 1 sell, never keep

Goat: 1 Kid: 1 sell always, never eat

Duck: 1 Eat, sell, eggs- eat and more chickens

Chicken: 4 Eat, sell, eggs- eat and more chickens

Pigeon (“Para”): 50 Eat, sell, eggs only for more pigeons

Ox: 0

“We rent tractor and driver Rs300 / Bigha. Price of ox is too high and with other business there is no one to look after ox. Tractor is easier and can do more work than an ox in little time.

If conditions were good we could buy oxen, but it is difficult to get a man to work them. Oxen are very useful, but because of money and man problem we are not buying them.”

In Assam: 1 Bigha = 100ft x 100ft 4 Bigha = 1 acre

**What is a ‘good’ animal?** How do you know?

Physique, length, size.

If cattle are short then legs are small, if body is thin then body will become long.

If long legs and round body- this is very good.

Ox- hump should be big.

**How do you know if an animal is healthy?** How do you know if an animal is unhealthy?

Stop eating, body hot, body shaking, high temperature, lay down, bad mood.

**What things limit how much your animals give?** ( provide for your household?)

**Young Animals**

Where do you keep your calves? Can we see please?

**How many of your cows/ buffs/ goats had a baby in the last year?**

1 cow had one calf

2 ox, 2 cow, 2 calf (1 born last year), 4 ducks.

**Where are those baby animals now?**

**When a calf is born, can you teach me about the first day of its life?**

(When should it first drink milk (colostrum)? How much? What can you do to help it to be healthy? Who helps it?)

Clean calf, help to stand, help to drink from mother- usually drinks after half an hour.

Phaa- ho: calf drinks first until full, then we milk, usually we get less than 1 litre, occasionally more than a litre if it is a very milky cow (17). We both do the same (16)(*Not sure of the veracity of this!*).

**How much milk does an older calf drink and how often does it get it?** (Can you teach me how you do this? Who is responsible for this?)

First milking at 10 -0 12 days- cow and calf together until then.

Separate calf at midnight, once calf is 3-4 months old- separate at 9 or 10 o’clock.

In morning calf drinks first for 10 minutes, then milk cow. We take less milk for the first month- 0.5L – then 1 – 1.5L daily after that. Milk once daily.

16- wife milks cow 17: husband and wife milk cow.

**Do your calves have any food apart from milk? Water?**( Where? How often? Who’s job?)

Both men same.

Calf eats food from 3- 4 months- eats cooked food with mother.

Calf starts to taste grass from 1 month. Some people milk the cow empty twice daily- then calf starts eating grass at 2 months- calf becomes more healthy and health of cow improves too.

Water 2 or 3 times daily

16: I am outside the village with business, my son and daughter work so my wife does all this kind of work.

17: Man and woman both do.

**How much milk does your family get each day from your animals?** (What do you do with it? When do you first milk a cow/ buff /goat for drinking?)

17: 0.5 – 1.5L daily / cow (depending on animal, stage of lactation) sell 1L /cow/ day =2L

16: ? (*wife tells husband*) drink it all, son drinks 0.5L daily, more if he can get it.

**Adult Animals**

**Can you show me where your animals rest in the day? At night?**

Day: paddy (“Patar”) –tie cow, calf free. Night: shed

Take to water lands (Kumud and Umesh)

**How do your animals get food? What**? (Who (family member) is responsible for this? Can we meet them please? How much land do you have? Can we see it?) **Where do your animals drink? How often?**

17: Grazing, rice straw (little given every day) cooked food- rice husk, pumpkin, banana, brinjal, bottle gourd, carrot

16: Rice husk, veges, cauli, aloo; rice straw

Flood time: keep most of straw for flood time, banana tree, if Forest Dept let us cut elephant grass feed that and weeds from water lands (“Dol- ghah” = elephant grass)

**Did you vaccinate your animals last year? Why / why not?**

17: No. Did only once- cow’s neck swelled but became well again, after that scared of vaccination

**Do you de-worm animals? How? Why/ why not?**

“Pel- lou” = worms. I never see any kind of worms in my cow dung so I never give medicine. I always give my animals salt and perhaps that is why I never see worms.

**Do you do anything else to help your animals?**

16: Keep shed clean every day. I light incense in there every day. Usually I wash my cattle, I wash my oxen after they cultivate, I wash my cows once a month. Mosquitos nest in dung.

17: “I take very good care of my cattle. If I see any kura phata in village I wash all my animals in hot water with salt or KMnSo_4_. Because of this I have not had Kura- phata in my cows for 8 years. I think only I do this, but sometimes I advise other people to do this as well. If you prevent in early days then disease does not have to spread to all animals. I advise other people to do this. If you cannot find KMSO_4_ then you must use salt.”

**You live close to the forest reserve. Does this affect you?**

17: “We mostly rely on crops. Crop raiding by elephants and wild boar is our most important problem. This is the main problem and nothing else. Sometimes elephants eat banana trees. It is in their nature, it is not a big problem. Forest guards do come to help us with thus problem. TCF have helped us providing tungi and torches. These have helped us to scare off elephants, we are very grateful to them.”

**Animal health education**

**Is there anyone who can treat your animals?** (What would you like the vet doctor to know more about?)

17: Dr comes 3.5km. He comes mostly when we call. He is a good Dr and I like him.”

**Would you like to learn more about animal health? Why?**

17: “I would like to learn more. It would be helpful to me.”

**What problems are there for people trying to learn about animal health in your village?** ( Why? Which ways of learning about animal health would be good for you (village meetings, classes, leaflets, radio broadcasts, internet information, etc)?)

17: “I know how to read and write in Assamese. If I got some posters and pamphlets I can learn from these. If photos are provided I can learn from these too but it should be in Asssamese. Practical training is also very useful because people can show me and I can learn by doing.”

**What changes could make your farm to more productive/ better?**

17: “I want to buy some high breed cows that can make more milk. I can afford to feed Jerseys which are cross breed with local cows, they need less food than pure bred cows.

**What do you think is the future of this farm? (**Who will you pass your knowledge of farming on to?)

16: The next generation

**Anything else you would like to tell us?**

*Umesh’s wife arrives*

“We are very grateful because you came and you teach us important things. We are very eargerly waiting to see you again.”

Umesh’s wife: “Nowadays the village is improving a little because we have a road to come here.”

“Usually we are doing crops in the winter time. The flood washes out every crop. Now we are doing the (un)seasonal crop because we have the water motors (pumps) and things are improvinhg bit by bit. Because of the flood and the crop raiding pattern we changed. Now the raiding pattern is changing too.”

Umesh

8.5 bihars: 2.5 Govt (farmed for many years), 6 rented + 3 bihars mustard.

If not raided- 3 bihars is enough to support us. I get 4 -5 quintals (1 quintal = 100kg) per bihar because I use less fertiliser and pesticide. Elephant takes more than 50% of crop. 2016 seasonal crop I got only 200kg from 4 bihars due to elephant raiding.

3 types of crop:

Hali: June – Nov/December

Bow: June – Nov / December (different type of rice)

Bodo- khati (irrigated crop): January – June (harvest before flood)

Seasonal crop- start little plants before flood, plant out after flood while rains still sufficient

“Khati” = cultivation

**17**

**Village**: DFP **Date**: **Time**:

Interviewer: Andy Translator: Jadu

**Location/ who else is present / what else is happening?**

In house, small (3?) daughter and mother in law present, men outside in yard.

**Name**: Lady and Mum in law **~~Male~~/ female:** **Age:** 25 + don’t know ( 50+)

**Your family and household**

**Who lives in your house?**

(Can we meet them please? What do they do? What do you want to do when grown up?)

Self

Husband 28 carpenter

Daughter 3

Sister in law 32

Brother in law 34 can do many jobs for money (handyman)

Brother in law 27 carpenter unmarried

Father in law

Mother in law

**How is your family/ household supported?** (food, money, rent, etc).

1. Carpentering
2. Farming- paddy (home), mustard (sell), veg- home and selling
3. Weaving- wants to expand, get sewing machines and get women working to make money through tailoring business.

**What animals does your family/ household have? Why are your animals important?** (Can we see your animals please? Why do you keep them? How do animals help to support your household? )

Cow: 2 milk, calves

Calf: 1 keep healthy female for home, always sell male after harvest approx. 1 – 1.5 y/o

Goat: 1 sell- castrate male (Dr does castration)

Duck: 5 selling, eating, eggs

Chicken: 30+ selling, eating, eggs

Ox: 0 Sold because of money crisis. Normally sell ox when old

“Use rented tractor. Father had a stroke, had to sell ox. Want to buy oxen now, then we won’t have to pay tractor. Ox is best, you can do whatever/whenever you want.”

**What is a ‘good’ animal?** How do you know?

Colour, health, physique. Ears erect, eyes (you know if animal is healthy by looking, legs- muscular, hoof- should be pointy, tight shape, upright, small gap.

“Colour- red and black mix, no white. White is not so good, not suitable for this home”

Cow: strong backside, if cow and calf, then look at calf.

“To buy (my father in laws’) sons all go together. Sometimes an experienced man from the village goes with them if available.”

“Saboo” = eyes

**How do you know if an animal is healthy?** How do you know if an animal is unhealthy?

Sad mood, stops eating, lays down, doesn’t want to go to graze.

**What things limit how much your animals give?** ( provide for your household?)

Lack of fodder, especially during flood.

**Young Animals**

Where do you keep your calves? Can we see please?

**How many of your cows/ buffs/ goats had a baby in the last year?**

2 cows, both calved

**Where are those baby animals now?**

1 here, 1 sold (we use calves as savings)

**When a calf is born, can you teach me about the first day of its life?**

(When should it first drink milk (colostrum)? How much? What can you do to help it to be healthy? Who helps it?)

Check navel, clean calf, check for wounds/ maggots ( do nothing if none found), if maggots present put Himax^TM^ or tobacco, may body bandage to colour.

Feed cow banana leaves (gol- phat) and warm water, especially if placenta did not pass.

Clean and tie rice straw in calf’s mouth (for maximum 30 minutes), calf then drinks for 10 – 30 minutes, calf is usually drinking 20- 30 minutes after born, then wash udder and milk out phaa-hoo, amount of phaa-hoo gained varies with udder size, usually about 1 litre.

**How much milk does an older calf drink and how often does it get it?** (Can you teach me how you do this? Who is responsible for this?)

Next milking at 10 -11 days, cow and calf together all the time until then. Milk cow once daily- separate calf from cow in the morning- milk cow 3 -4 hours later- calf drinks for 5 minutes- then milk. Get 1 litre / cow per day. (2 cows therefore 2 litres)

**Do your calves have any food apart from milk? Water?**( Where? How often? Who’s job?)

Start eating at 2 – 3 months old. Grazing plus cooked food with mother.

Take water with mother cow.

Mother in law is main cow carer, she milks and takes water. Other family members help if available.

**How much milk does your family get each day from your animals?** (What do you do with it? When do you first milk a cow/ buff /goat for drinking?)

1 L/ cow / day = 2L usually use one and sell one.

**Adult Animals**

**Can you show me where your animals rest in the day? At night?**

Paddy in day- tie cow, calf loose, shed at night

**How do your animals get food? What**? (Who (family member) is responsible for this? Can we meet them please? How much land do you have? Can we see it?) **Where do your animals drink? How often?**

Water 3x daily- mother in law does

Cooked food: chopped banana tree and veg, occasionally cow food mix.

In flood- rice straw, banana tree and leaves.

**Did you vaccinate your animals last year? Why / why not?**

Yes. For at least 5 years. Mother in law knows why, young lady also knows why- it’s about preventing Dz- especially saboka.

**Do you de-worm animals? How? Why/ why not?**

Give medicine- adult cows at 1 year. Never to calf or goats.

**Do you do anything else to help your animals?**

Vitamins sometimes.

**You live close to the forest reserve. Does this affect you?**

**Animal health education**

**Is there anyone who can treat your animals?** (What would you like the vet doctor to know more about?)

Dr- Saboo- 4km away. He comes when called. I like the Dr.

**Would you like to learn more about animal health? Why?**

Yes. If I know more about cattle I can produce more.

**What problems are there for people trying to learn about animal health in your village?** ( Why? Which ways of learning about animal health would be good for you (village meetings, classes, leaflets, radio broadcasts, internet information, etc)?)

“If someone explains in a good way I can learn from meetings. Also I can learn from practicals.” (young lady)

“Yes, I can learn from leaflets, but day by day my eyesight is dropping. Dr told me to wear glasses but I am not doing.” (old lady)

**What changes could make your farm to more productive/ better?**

“Buy high quality hybrid cows to increase production. I think I can produce for most of the year, but there will not be sufficient fodder in flood time. That is why I am not buying (cows).”

“I think a lot, that’s why my hair is going white.” (old lady)

**What do you think is the future of this farm? (**Who will you pass your knowledge of farming on to?)

“Son, and then I don’t know, I can’t predict the future.”

**Anything else you would like to tell us?**

“I am very tranquil, but I don’t understand everything you said.” (old lady)

“I do want to learn more about animal health. I am very grateful to meet you, I am hoping to see you again in the future.”
