# Supplementary material for: Colostrum provision and care of calves among smallholder farmers in the Kaziranga region of Assam, India
Source: PLoS One. 2020 Mar 11;15(3):e0228819. doi: 10.1371/journal.pone.0228819 (PMC7065800; doi:10.1371/journal.pone.0228819)
Supplement: Attachment — Submitted filename: Response to reviewers.docx [file pone.0228819.s002.docx]

Dear Arda Yildirim and Reviewers

Thank you for the time taken to review our manuscript and for your positive and constructive comments.

We have attempted to address all of the points that you have raised, please find these detailed in an item by item fashion below

Please ensure that your manuscript meets PLOS ONE's style requirements, including those for file naming. The PLOS ONE style templates can be found at

This has been altered in accordance with the templates provided. Thank you.

2. Your ethics statement must appear in the Methods section of your manuscript. If your ethics statement is written in any section besides the Methods, please move it to the Methods section and delete it from any other section. Please also ensure that your ethics statement is included in your manuscript, as the ethics section of your online submission will not be published alongside your manuscript.

The ethics statement now appears beneath a separate subheading in the methods section.

3. We note that Figures [1 and 4] includes an image of a participant in the study.

The narrative of the manuscript is of the co- existence and co- dependency of people and domestic animals, and we believe that the manuscript is greatly enriched for the addition of relevant images to illustrate this. These two images have been specifically chosen as not only do they illustrate key points, the human participants are impossible to identify as no details of faces are visible whatsoever. The individuals concerned did supply informed verbal consent, which was recorded, however, for the reasons explained in the manuscript written consent was not sought. We would request that as one cannot identify the individuals shown in the images you would consider accepting these images for publication. If it remains impossible to use these images, we will be able to alter the manuscript to include others, however we have specifically selected these images for their contribution to the richness of the manuscript, while preserving the anonymity of the participants. Thank you.

Reviewer #1: This is an interesting study. As someone who does work in India, I commend you for the time it would take to conduct this type of evaluation of calving and calf management practices. And as someone that works on calves, this is also interesting.

There are a few spelling and grammar mistakes throughout, but overall it is well written.

Thank you for your kind and constructive comments. We have revised the manuscript to remove all errors that we have found and to improve the clarity of written English.

Please exchange may for might. The use of may implies asking permission, whereas something might happen.

We have amended the use of the word “may” throughout the manuscript to use terms such as “might”, “can” or “sometimes”. Thank you for bringing the need for these changes to our attention as this improves the clarity of the manuscript.

Line 60: This is true only as an example. IgG levels can vary greatly due to the nutrition, environment and immunological status of the dam, so it is imperative in any system to have some way to measure immunoglobulins on a cow by cow basis. Please make a statement about that and there are many references in the literature.

We have added a statement regarding factors resulting in variation in colostrum quality and a second statement on the value and challenges of assessing individual animals with supporting references. Thank you for raising this important point.

264 start sentence with something other than a number.

Done. Thank you.

Reference out of place

Attended to. Thank you.

It would be helpful if you made a suggestion about what type of intervention and educational materials should be provided. Some of the farmers/people are illiterate from my experience which means educating them about science requires a different format of information exchange. What would you recommend?

We have briefly expanded upon this point at the end of the discussion, with particular reference to a previous project carried out in Madhya Pradesh. Currently we are trialling an intervention in the locality, which will be analysed and reported next year.

Reviewer #2: This manuscript is interesting and useful to develop and plan regional dairy farm/industry. However, the author should present their results in a rather more objective manner. for example inadequate, notable, commonly practiced etc are not scientific wording. In addition, the author need to clarify types of cows.

Thank you for your positive and constructive comments.

The manuscript has been revised to replace these terms, and others, with more precise language. Thank you for bringing this to our attention as these changes have improved the manuscript.

Local cows are described in the manuscript line 215 – 217. The types of cows owned by the participants are now explicitly stated in the relevant section of the results, line 251- 253.

We hope that the revised manuscript now meets with your approval for publication. Should further alterations be required, please do not hesitate to contact us further.

Yours sincerely

Andy Hopker
